# The Prevalence of Allergic Rhinitis, Allergic Conjunctivitis, Atopic Dermatitis and Asthma among Adults of Tehran

**Published:** 2018-11

**Authors:** Raheleh SHOKOUHI SHOORMASTI, Zahra POURPAK, Mohammad Reza FAZLOLLAHI, Anoshirvan KAZEMNEJAD, Fatemeh NADALI, Zahra EBADI, Behnoosh TAYEBI, Majid MOSLEMI, Akram KARIMI, Shahnaz VALMOHAMMADI, Amir Masoud NAZEMI, Adriano MARI, Mostafa MOIN

**Affiliations:** 1. Immunology, Asthma and Allergy Research Institute, Tehran University of Medical Sciences, Tehran, Iran; 2. Dept. of Biostatistics, Faculty of Medical Sciences, Tarbiat Modares University, Tehran, Iran; 3. Dept. of Hematology and Blood Transfusion, School of Allied Medical Sciences, Tehran University of Medical Sciences, Tehran, Iran; 4. Blood Transfusion Organization Research Center, Tehran, Iran; 5. Ibne Sina Clinic, South Hygiene Center, Tehran University of Medical Sciences, Tehran, Iran; 6. Associated Centers for Molecular Allergology, CAAM, Rome, Italy; 7. Dept. of Immunology and Allergy, Children Medical Center, Tehran University of Medical Sciences, Tehran, Iran

**Keywords:** Prevalence, Allergic rhinitis, Allergic conjunctivitis, Atopic dermatitis, Asthma

## Abstract

**Background:**

Alteration of environmental factors and air pollution affects the trend of allergic diseases especially in cities such as Tehran. This study aimed to determine the prevalence of allergic rhinitis, conjunctivitis, atopic dermatitis and asthma among adults in the capital city of Iran.

**Methods:**

This cross-sectional study was performed between 2013 and 2016 in Tehran, Iran. The participants were adults between 18 and 45 yr of age. A specific questionnaire including demographic data and clinical symptoms was filled out by a trained interviewer. The diagnosis of allergic diseases was performed based on standard questionnaires and criteria.

**Results:**

The prevalence of allergic rhinitis, conjunctivitis, asthma and atopic dermatitis were 28.3%, 15.9%, 7.6% and 3.9%, respectively. Allergic rhinitis and conjunctivitis together were reported in 12.3% of the participants. Among patients with asthma, 47.4% had AR. Additionally, 25.7% of atopic dermatitis subjects were reported to have asthma. The subjects with at least one of these allergic diseases were 36.3%. Women showed a higher prevalence of allergic symptoms than men. There was a significant relationship between allergic symptoms and family history of atopic diseases.

**Conclusion:**

The most common allergic disease was allergic rhinitis. Regarding the comorbidity of asthma and allergic rhinitis, paying more attention to controlling these allergic diseases is deemed necessary.

## Introduction

The increased prevalence of allergic diseases has been reported in different epidemiologic studies, which could be due to environmental changes (1). This increase should be considered as a public health problem worldwide. Hundreds of millions suffer from allergic rhinitis and asthma (2).

Different organs can be affected by allergic diseases (1) the most common of which are asthma, allergic rhinitis and atopic dermatitis (3). These diseases result in the inability and ailment of the patients (4), affecting their quality of life, making them unable to work or study (5, 6) and imposing a significant socioeconomic burden on families and the health care system (4).

Air pollution, climate change, and global warming can affect the trends of allergic diseases. They do so by giving rise to the growth of plants through increasing, precipitating or prolonging pollination (7). There is a positive relationship between living in urban areas and air pollution on the other hand and the rise of allergic diseases (especially respiratory allergies) on the other (7, 8). Tehran, the capital of Iran with around 9 million inhabitants, has severe air pollution problems and the health of its inhabitants has seriously been affected by different diseases especially cardiovascular and respiratory disorders (9).

There are very few epidemiological studies on the prevalence of allergic diseases in adults. Studies in Tehran (10), Mashhad (11) and Urmia (12), have reported the prevalence of asthma or allergic diseases among the adult population. Therefore, taking into consideration the data of a number of studies and the increase in air pollution and environmental changes in Tehran, this study aimed to determine the prevalence of asthma, allergic rhinitis, allergic conjunctivitis and atopic dermatitis among adults in Tehran from 2013 to 2016.

## Materials and Methods

This cross-sectional study was conducted between 2013 and 2016. The participants were adult volunteers aged 18–45 from the 22 districts of Tehran, Iran referred to Iranian Blood Transfusion Organization and Tehran University of Medical Sciences. Based on the inclusion criteria, participants had to be between 18 to 45 yr of age and had to be a resident of Tehran during the last year.

This study was approved by the Ethics Committee of Immunology, Asthma and Allergy Research Institute. A signed informed consent was obtained from all participants before the interview.

In addition to questions related to clinical symptoms, demographic data (age, gender, and education), smoking behavior and family history of allergic diseases were also recorded. The questionnaires were filled out by a trained interviewer.

### Definitions and Questionnaires

This study’s questionnaire included 4 sections: questions about allergic rhinitis and conjunctivitis (from English version of Tuohilampi) (13), asthma (European Community Respiratory Health Survey, ECRHS) (14), and atopic dermatitis (UK Diagnostic Criteria for AD) (15). The questionnaire was translated into Persian. Then, it was translated back to English. The validity was confirmed by an expert committee including one immunologist, five subspecialists in allergy and clinical immunology and one biostatistics. The reliability was determined by kappa coefficient.

The ECRHS questionnaire was used to determine the prevalence of asthma symptoms (14). The ECRHS questions related to asthma included wheezing in the last 12 months, breathlessness with wheezing, waking up with tightness, shortness of breath and coughing in the last year, having asthma attacks, and medication for asthma symptoms. Positive response to questions including “wheezing and wheezing accompanied by breathlessness” was considered in the definition of asthma (16).

The English version of Tuohilampi questionnaire was used for allergic rhinitis and allergic conjunctivitis (AC). The questions inquired respondents about “(a) chronic nasal symptoms without cold, (b) allergic nasal symptoms from pollen or animals and (c) Physician Diagnosis of AR” (13). Having nasal allergic symptoms resulting from pollen or animals was considered as the criterion for AR (13).

The allergic conjunctivitis questions asked the samples whether they had “(a) irritation of the eyes without a cold, (b) allergic eye symptoms resulting from pollen or animals and (c) Physician Diagnosis of AC”. The positive response to eye allergic symptoms resulting from pollen or animals was considered as the criterion for AC (13).

The UK diagnostic criteria were used to evaluate AD. The questions inquired the respondents to see if they ever had “itchy skin, history of flex-uraleczema, generalized dry skin, history of asthma or AR, onset of rash under the age of two and visible flexural dermatitis”. Itchy skin along with three other mentioned symptoms was considered as the diagnostic criterion for AD (15).

### Statistical Analysis

The data were analyzed by SPSS ver. 20 (Chicago, IL, USA). Kappa coefficient was calculated for evaluation of reliability. The frequency and percent of categorical variables were calculated. Chi-squared test was used for relationship between two categorical variables. Odds ratio was determined to evaluate the association between two categorical variables. *P*-value less than 0.05 was considered as significant. The graph was drawn using Prism 5 (Graphpad Software Inc., La Jolla, CA, USA) software.

## Results

In this cross-sectional study, 2569 subjects were included. The mean and standard deviation of age were 30.72± 7.06 yr. The participants were 1866 males (72.6%). The demographic characteristics of participants are summarized in Table 1. The questionnaire’s kappa coefficient of agreement was between 0.7 and 1.

**Table 1:** The demographic characteristics of study participants

***Variables***	***N (%)***
Sex	
Male	1866(72.6)
Female	703(27.4)
Age Groups(yr)	
18–25	662(26.2)
26–35	1190(47)
36–45y	678(26.8)
Smoking Status	
Non-Smoker	2014(79.7)
Smoker	424(16.8)
Ex-Smoker	40(1.6)
Hubble bubble	50(2)
Education	
Under diploma	162(6.4)
Diploma	810(32.2)
BS	1158(46)
MS	269(10.7)
PhD	116(4.6)
Family History of Allergic diseases	
Yes	697(28.1)
No	1785(71.9)

A significant difference was observed in the frequency of allergic symptoms (except wheezing) between males and females. Women showed a higher prevalence of allergic symptoms (Table 2).

**Table 2:** The frequency of allergic symptoms according to sex in Tehran

	***Allergic Symptoms***	***Males***	***Females***	***Total***	***P value***
		***N (%)***	***N (%)***	***N (%)***	
Asthma	Wheezing	311(16.7)	115(16.4)	426(16.6)	0.85
	Wheezing with Breathlessness	115(6.2)	79(11.3)	194(7.6)	<0.001
	Wheezing without cold	170(9.1)	70(10)	240(9.4)	0.51
	Chest Tightness	60(3.2)	91(13)	152(5.9)	<0.001
	Shortness of breath	83(4.5)	67(9.5)	150(5.8)	<0.001
	Nocturnal cough	107(5.7)	118(16.8)	225(8.8)	<0.001
	Asthma Attack	15(0.8)	20(2.8)	35(1.4)	<0.001
	Medication for asthma	48(2.6)	29(4.1)	77(3)	0.04
	Chronic nasal symptoms without cold	501(26.9)	246(35)	747(29.1)	<0.001
	Allergic nasal symptoms from pollen or animals	502(27)	224(32)	726(28.3)	0.01
AR	Physician Diagnosis AR	249(13.4)	147(20.9)	396(15.4)	<0.001
	Irritation of eyes without cold	358(19.2)	234(33.3)	592(23.1)	<0.001
	Allergic eye symptoms from pollen or animals	243(13.1)	164(23.4)	407(15.9)	<0.001
AC	Physician Diagnosis AC	105(5.6)	78(11.1)	183(7.1)	<0.001
	Itchy skin	248(13.3)	275(39.2)	523(20.4)	<0.001
	FlexuralEczema	41(2.2)	68(9.8)	109(4.3)	<0.001
	Generalized dry skin	44(2.4)	114(16.4)	158(6.2)	<0.001
AD	Rash under 2 y	11(0.6)	50(7.2)	61(2.4)	<0.001
	Visible flexural dermatitis	23(1.2)	42(6.1)	65(2.6)	<0.001

AR=Allergic Rhinitis, AC=Allergic Conjunctivitis, AD: Atopic Dermatitis

A positive family history of allergy was found in 28.1% of all participants while 42.2% of allergic subjects had positive family history. There was a significant relationship between allergic symptoms with a family history of allergic diseases (*P*<0.001, OR=2.90).

The prevalence of at least one of the allergic diseases was found in 36.3% of the cases. The most prevalent allergic diseases were AR and AC (28.3% and 15.9%, respectively). Among subjects with AR, the frequency of AC and asthma were, in turn, 43.6% and 12.5%. Additionally, 12.3% of the study population reported to have AR and AC at the same time.

**Fig. 1:**
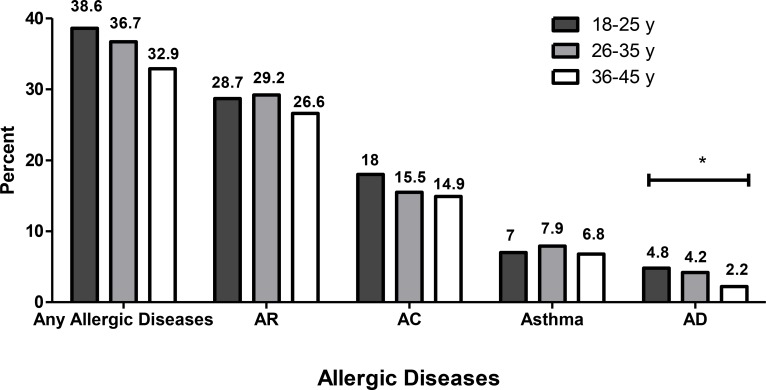
The prevalence of different allergic diseases according to age groups. AR=Allergic Rhinitis, AC=Allergic Conjunctivitis, AD: Atopic Dermatitis. (*P<0.05)

The prevalence of wheezing and asthma were 16.6% and 7.6%, respectively. Among cases with asthma, 47.4% had AR. In addition, 13.4% of the subjects with asthma verified that they also had atopic dermatitis. There is a significant association between smoking habit and some asthma symptoms including wheezing, wheezing with breathlessness and nocturnal shortness of breath (*P*<0.001). Moreover, 3.9% of the subjects had AD. The frequency of asthma was 25.7% in participants with atopic dermatitis. The prevalence of AD was statistically different between age groups (*P*=0.03). With increase in age, the prevalence of AD plummeted (Fig. 1).

Eight subjects (0.3%) had all diseases at the same time. The statistical analysis revealed a significant relationship between the educational level and the prevalence of AR, AC and AD (*P*<0.001). In subjects with higher educational levels, the prevalence of allergic rhinitis symptoms was more frequent.

## Discussion

The prevalence of at least one of the allergic disease was 36.3% among the adult’s population. The most common allergic disease was allergic rhinitis (28.3%) followed by allergic conjunctivitis (15.9%). Interestingly, in Korea, the overall prevalence of allergy was reported 37.6% in subjects more than 10 yr old (17).

Consistent with our study, in a telephone survey in Tehran, the prevalence of AR was 26.7% (18). In Mashhad, the lower prevalence of AR reported (22.4%) compared to our study (11). The results of our study regarding AR are somehow similar to those of Italy (25.8%) (19) and Turkey studies (23.1%) (20) while in the UAE, the prevalence of AR symptoms was shown to be 7% in all ages (21). In contrast to our study, a higher prevalence of current rhinitis was reported in Sweden (42.8%) (22).

AC was reported by 15.9% of our participants as a common allergic problem; afflicting 6% to 30% of the world population (23). This disorder is usually manifested in 50% to 70% of AR patients (24) the prevalence of which was 43.6% in this subgroup of subjects in the current study. The allergic conjunctivitis prevalence is not often determined because most of the questionnaires do not include questions about allergic conjunctivitis symptoms and ocular problems are often assessed by different specialists (23).

The prevalence of asthma was reported 7.6% in this study. To our knowledge, there has not been any recent study on asthma prevalence in Iranian adults since 2013. The estimation of asthma prevalence is complicated because of different meanings of asthma symptoms and current asthma (25). In a telephone survey in Tehran, in adults aged 20–44, the prevalence of wheezing (24%) and asthma (wheezing and breathless) (10.8%) was reported to be more than those of our study (10). In Urmia and Mashhad, the prevalence of most asthma symptoms was less than that of our study (11, 12).

There are many reports about the frequency of asthma symptoms throughout the world. The mean frequency of wheezing and physician-diagnosed asthma among 70 world counties was 8.6% and 4.27%, respectively. China (1.73%) and Australia (27.4%) showed the minimum and maximum wheezing frequency in their population. Australia, England and Netherland showed the highest prevalence of asthma symptoms. Turkey, Pakistan and the UAE as Iran’s neighboring countries, in turn, showed 11.34%, 5.02% and 7.21% wheezing (6). In Canada, the prevalence of wheezing and breathlessness with wheezing was higher than the figures cited in our study. Furthermore, females showed higher frequency than males (26). Our findings showed a higher prevalence of wheezing (16.6%) in comparison with most Asian countries and the lower frequency of wheezing compared to some European countries and Canada (6, 26).

Many patients with asthma experience AR, which is an important comorbid with asthma (27). In the present study, 47.4% of participants with asthma symptoms had AR. Additionally, 12.5% of AR cases asserted that they exhibited asthma symptoms.

In this study, 3.9% of participants had AD. The result of this study was less than the one reported in Mashhad (6.6%) (11). Some countries such as Colombia (11.45%) (28) had a higher prevalence. In Europe, the prevalence of AD was 6.4% in men and 8.01% in women. Similar to our study, females were cited to have a higher prevalence. Being a female can increase odd ratio of developing AD (29).

In most studies including our study, adult females indicated a further prevalence of asthma and allergic symptoms compared with males. The cause of this is attributed to a rise in sex hormones such as progesterone (pro-inflammatory) in females and testosterone (anti-inflammatory) in males (30) and other factors including obesity and different environmental exposure (31).

Different factors could contribute to reporting the different prevalence of allergic diseases including geographical and ecological factors, genetic and ethnic variations, lifestyle differences (25), the methods or questionnaires employed for evaluation, different age groups and the time when a study is conducted (29).

Tehran as one of the most polluted cities in the world contains many air pollutants in its atmosphere. These air pollutants result in a variety of health issues (9). The airway mucosal tract could be injured by air pollution so aeroallergens could have easier accessibility to immune cells and increase respiratory sensitization. Due to high levels of air pollution in big cities (such as Tehran), allergic diseases are expected to rise (32). The prevalence of asthma and AR symptoms is higher in Tehran than in many other Asian countries (6, 20, 21).

In addition to providing updated data on prevalence of asthma and AR, the strength of this cross-sectional study is that it is the first of its kind to have determined prevalence of AC and AD in Tehran’s adults. The limitation of our study was the low number of female participants in the study have biased the overall allergic disease prevalence.

## Conclusion

The prevalence of allergy was 36.3% in our study. Although the prevalence of AC and AD were 15.9 and 3.9%, respectively, the most common allergic diseases was AR (28.3%) which is evidence showing somehow a high prevalence and being considered as a comorbid with asthma (12.5%). With respect to these results, imperative to pay more attention to preventing and treating allergic diseases in adults.

## Ethical considerations

Ethical issues (Including plagiarism, informed consent, misconduct, data fabrication and/or falsification, double publication and/or submission, redundancy, etc.) have been completely observed by the authors.
